# Kinases: Understanding Their Role in HIV Infection

**DOI:** 10.4236/wja.2019.93011

**Published:** 2019-09-09

**Authors:** William De Martini, Roksana Rahman, Eduvie Ojegba, Emily Jungwirth, Jasmine Macias, Frederick Ackerly, Mia Fowler, Jessica Cottrell, Tinchun Chu, Sulie L. Chang

**Affiliations:** 1 Department of Biological Sciences, Seton Hall University, South Orange, NJ, USA; 2 Institute of NeuroImmune Pharmacology, South Orange, NJ, USA

**Keywords:** HIV, AIDS, Kinases, IPA, NF-κB

## Abstract

Antiviral drugs currently on the market primarily target proteins encoded by specific viruses. The drawback of these drugs is that they lack antiviral mechanisms that account for resistance or viral mutation. Thus, there is a pressing need for researchers to explore and investigate new therapeutic agents with other antiviral strategies. Viruses such as the human immunodeficiency virus (HIV) alter canonical signaling pathways to create a favorable biochemical environment for infectivity. We used Qiagen Ingenuity Pathway Analysis (IPA) software to review the function of several cellular kinases and the resulting perturbed signaling pathways during HIV infection such as NF-κB signaling. These host cellular kinases such as ADK, PKR, MAP3K11 are involved during HIV infection at various stages of the life cycle. Additionally IPA analysis indicated that these modified host cellular kinases are known to have interactions with each other especially AKT1, a serine/threonine kinase involved in multiple pathways. We present a list of cellular host kinases and other proteins that interact with these kinases. This approach to understanding the relationship between HIV infection and kinase activity may introduce new drug targets to arrest HIV infectivity.

## 1. Introduction

The Human Immunodeficiency Virus (HIV) is a member of the *Retroviridae* family that adversely affects the immune system which can lead to acquired immunodeficiency syndrome (AIDS) [1]. The contact of bodily fluids such as blood, semen, and vaginal fluids with mucous membranes or directly to the blood stream by needles provides successful transmission of HIV [2]. According to the CDC, there are three stages of HIV infection. The first stage is acute HIV infection where the patient is the most contagious. It involves a flu-like illness that usually starts about 2 weeks after infection. The second stage is named clinical latency. This nomenclature for this stage is misleading as the virus is actively replicating and there is declination of CD4+ count from 30 to 60 cells/mm^3^/year [3]. The infected person will experience little to no symptoms during this time period, which can last decades with proper treatment. The last stage is acquired immunodeficiency syndrome, or AIDS [4]. This stage occurs when the immune system is so badly damaged that the infected person contracts multiple opportunistic infections such as *Molluscum contagiosum, Toxoplasma gondii* encephalitis, cytomegalovirus-associate retinitis, non-Hodgkin’s lymphoma and Kaposi’s sarcoma [1] [5]. Once this stage is reached, the typical life expectancy is about three years without treatment [2].

HIV is believed to have originated from chimpanzees through the simian immunodeficiency virus (SIV) to humans in Africa between 1884 and 1924, and it entered the United States around 1970 [6]. The HIV epidemic began in the United States in June 1981, when 26 homosexual men were diagnosed with Kaposi’s sarcoma, which was generally only found in older immunosuppressed patients [7]. In late 1982 and early 1983, unexplained immunodeficiency in both infants and female sexual partners of men with AIDS was discovered in the United States [7]. During this time HIV (HIV-1) was isolated at the Insitut Pasteur from lymph node tissue of an individual with generalized lymphadenopathy [8]. However, because the time between infection with HIV and the onset of AIDS can be as much as fifteen years, it was hard to prove that HIV was the cause of AIDS [7]. By 1985, AIDS had been identified in all regions of the world [6].

### 1.1. Statistics and Epidemiology

In studies by UNAIDS in 2013, the number of adults and children living with HIV steadily rose from 2000 to 2012, while the number of AIDS related deaths and new infections of HIV steadily declined over the twelve year span. This can be attributed to organizations such as UNAIDS increasing knowledge about HIV, as well as there being a higher distribution of antiretroviral drugs to these lower income areas. In 2004, there was a spike in HIV infections and AIDS related deaths, but there has been a global decline in both ever since and is predicted to stay on this decline without any further spikes [9]. In addition to geographical location being related to HIV incidents and AIDS related deaths, certain populations are also at a higher risk for infection including, men who have sex with men, transgender people, people who inject drugs and sex workers [10]. According to the World Health Organization, as of 2018 there were 36.9 million people living with HIV with 59% of them receiving antiretroviral treatment. Out of these 36.9 million people, 25.7 million of this large group of HIV infected individuals were from Africa with 60% of the population receiving antiretroviral therapy. Populations in South East Asia and the Eastern Mediterranean had the lowest amount of people receiving antiretroviral treatment with 51% receiving treatment in Southeast Asia and 18% receiving treatment in Eastern Mediterranean [9].

### 1.2. Mechanism of HIV Infection

HIV is transmitted when an infected individual’s bodily fluids (ex. blood, semen) comes into contact with another individual’s mucous membranes or damaged tissue. Once infected, HIV infects CD4+ lymphocytes, white blood cells used to help the body defend against pathogens [5] [11]. As a result the individual is vulnerable to infection by other foreign organisms, and subsequently may lead to sickness. HIV does this by first attaching itself to the CD4+ receptor on the CD4+T lymphocyte, and then binding to either the CCR5 or CXCR4 core-ceptor. The virus fuses its viral envelope with the cell membrane of the CD4+ T lymphocyte, allowing it to enter the cell. Once the virus is inside the CD4+ T lymphocyte it uses reverse transcriptase to make copies of its genome from DNA. Due to a lack of proofreading function by HIV reverse transcriptase mutations prevents antiretroviral drugs from working efficiently [1]. After making copies of its genome and converting it to DNA the HIV virus is able to enter the cell nucleus and incorporate itself to the genetic material. HIV integrates into the host genome through the integrase enzyme packaged in the virion during infection. The white blood cell then replicates the CD4+ T lymphocyte DNA with the integrated HIV DNA, and starts to produce long chains of HIV proteins which are used for further production of HIV virions [1]. These HIV proteins travel to the cell surface of the host with the two copies of the HIV genome and then assemble into noninfectious HIV viruses. Once these immature viruses are assembled, the budding process of virus particles occurs. HIV protease breaks up the protein chains that formed with the HIV genome by breaking the long protein chains down so that the smaller protein pieces of HIV are able to come together to create a fully mature, infectious HIV virus particle. From here, these viruses are now able to infect other CD4+ T lymphocyte and multiply, further affecting and damaging the individual’s immune system [11].

HIV-1 expresses multiple regulatory and accessory proteins such as trans-activator of transcription (Tat), negative regulator factor (Nef) and virion infectivity factor (Vif) during viral infection to create a favorable cellular environment for the lifecycle. Tat is an early regulatory protein necessary for replication as its loss of function results in no production of new virions [1] [3]. Tat is required for NF-κB dependent HIV-1 LTR activation [12]. Another early protein expressed in HIV-1 life cycle Nef which downregulates the expression of the CD4 receptor. Nef is able to induce T-Cell activation and NF-κB activation leading to increase expression of Tat [13]. Vif is expressed late in the HIV-1 lifecycle which is necessary for replication in cell line such as lymphocytes and macrophages and inactivates the antiviral activity of cytidine deaminases [1] [3].

### 1.3. AIDS Therapy and Treatments

Currently there are very few FDA-approved therapies for people infected with HIV in the United States, however there are many potential treatments on the way. When discussing HIV, the US Department of Health and Human Services only focuses on antiretroviral therapy (ART) as a treatment for those who have been infected by HIV [14]. Antiretroviral therapy is a specified regimen of drugs used to significantly decrease a person’s viral load which allows them to lead a somewhat normal life [14]. The use of ART allows the body to continuously maintain an undetectable viral load making HIV non-transmissible from that person [14]. Granted as technology improves scientists have been able to come up with other promising treatment/prevention plans. A promising prevention plan, which has been used in a clinical trial, utilizes the cell that is capable of secreting antiviral proteins (AVPs). AVPs have the capability to defend HIV target cells that have not been previously altered [15]. These findings are hopeful because both hematopoietic and non-hematopoietic cells can release AVPs allowing for all cell types to be reached [15]. A potential treatment for HIV also involves the use of hematopoietic cells, specifically RNA-based hematopoietic cells [16]. This gene therapy method has seen more interest since the recent improvements in understanding both RNA interference (RNAi) and microRNAs (miRNA) however no significant results have been found yet [16]. Another potential HIV therapy is genome editing (GE), which is similar to gene therapy, but is used to specifically alter cell genomes that are infection-related [17]. In recent studies of GE-based HIV therapies, CCR5 is used as the target gene of change due to its similarity to the CCR5δ32 genotype which is a naturally occurring HIV resistance gene [17]. Although many of the HIV therapies and prevention methods have not yet been approved, researchers globally continue to explore new methods to ensure safety in HIV therapeutics.

### 1.4. The Role of Kinases in Biology

Protein kinases operate as a regulatory species of enzymes that can modify the function of a target protein or enzyme substrate. Kinases can regulate substrate function via phosphorylation, a type of post translational modification. Phosphorylation is the addition of a phosphate group onto a free hydroxyl on the side chain of an amino acid residue. This post translation modification can increase or decrease the protein or enzyme substrate activity and is critical in many signal transduction pathways such as metabolism, transcription, cell cycle progression, cytoskeletal rearrangement, differentiation, cell movement, intercellular communication and more [18]. In mammalian cells three specific amino acids can be phosphorylated; serine, threonine, and tyrosine, thus a catalytic species of protein kinases exist for each of these target amino acid residues within the substrate. Serine kinase, threonine kinase, tyrosine kinase, and dual specificity kinases utilize ATP as a source for monophosphate; however, ATP is thermodynamically stable in triphosphate configuration, therefore kinases have several key domains to successfully capture, hold, and strip ATP of its gamma phosphate group for the phosphorylation of their target substrate [19].

The first key domain of a protein kinase is the ATP binding pocket; which, consists of a two specific amino acid sequences to hold ATP within the kinase. The glycine rich sequence loop that holds onto ATP from the top, and the conserved lysine sequence holds ATP in place; the next region of the ATP binding pocket is the conserved glutamate sequence, which stabilizes the kinase’s structure via a salt bridge formation with the conserved lysine sequence. Morphologically the adenosine of the ATP molecule is buried in the hydrophobic portion of the pocket while the terminal phosphate is directed towards the solution [19]. Mutations within the ATP binding pocket prevent kinases from phosphorylating their substrate because a lack of ATP binding to the kinase.

The second key domain of protein kinase is the substrate binding region. In this region of the kinase the activation loop is a specialized sequence that recognizes specific amino acid residues on the surface of the substrate. Allowing the kinase to distinguish its target residue; this is the only variable region within all classes of protein kinases. The last key domain of a protein kinase functions to transfer the phosphate group from ATP to the substrate utilizing two key motifs. The DFG motif binds a metal cofactor to cleave the phosphate group from ATP. Subsequently the HRD motif transfers the cleaved gamma phosphate from ATP onto the substrate via a catalytic aspartate residue found in this motif [20].

Phosphorylation caused by protein kinases is a reversible post translational modification in which protein phosphatases remove this phosphate group added, making it rapid and highly efficient. Kinases can also be easily regulated by other kinases such as the example of mitogen-activated protein kinase kinase (MAPKK) phosphorylating mitogen-activated protein kinase (MAPK). The significance of kinases can be demonstrated with the fact that over 500 protein kinases are encoded in the human genome and about 40% of all proteins are phosphorylated [21].

## 2. Host Cell Kinases and HIV Infection

**Figure 1.**
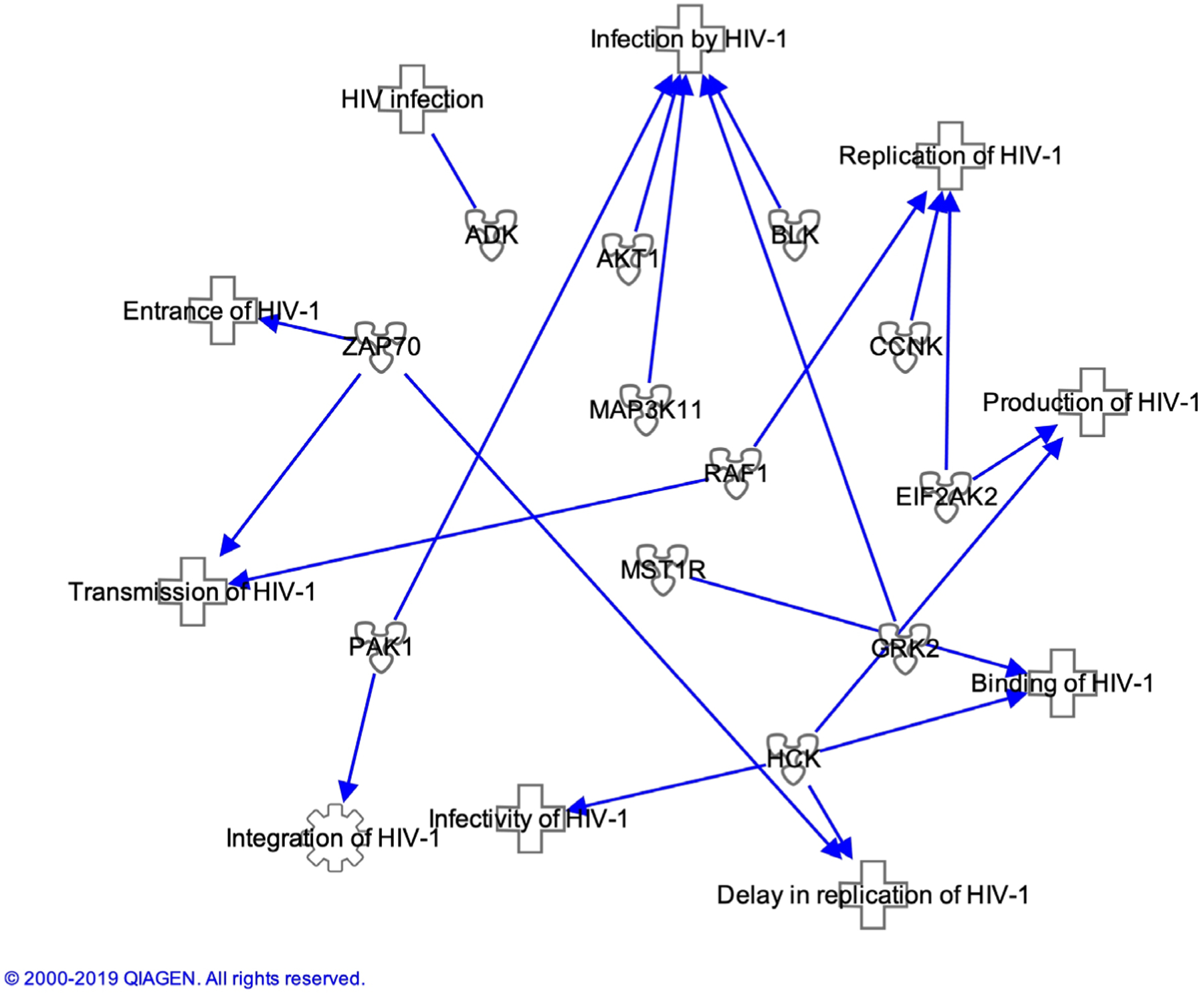
Top 12 Kinases involved in HIV Infection. A query was performed in IPA software to generate a list of all molecules known to be involved in HIV infection. Seventy of 1783 molecules discovered are kinases with the top 12 kinases represented in this figure.

**Figure 2.**
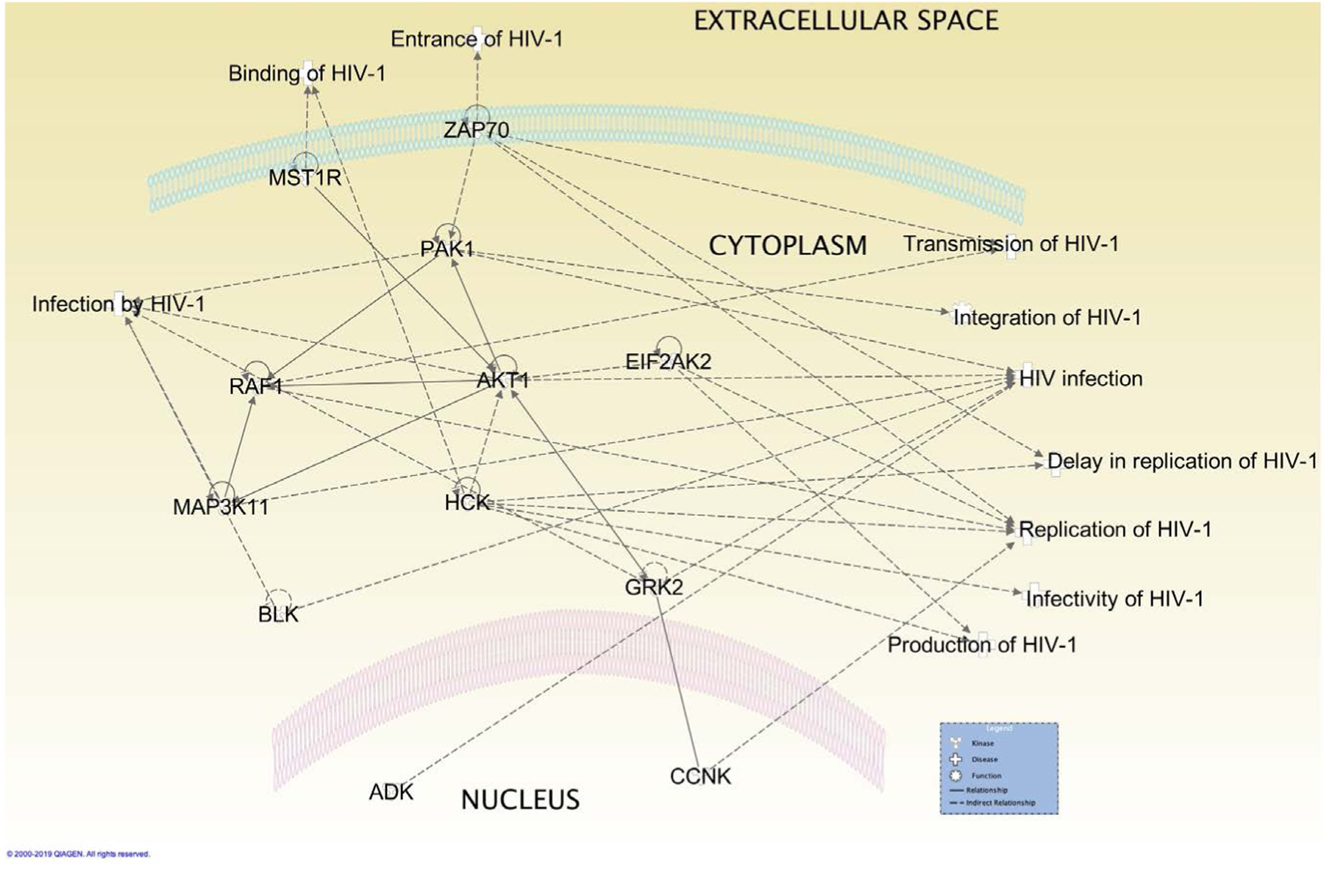
Location and interrelationship of top 12 kinases in HIV infection. Host cell kinases involved in HIV are located in various parts of the cell and influence the activity of one another. The dashed lines represent indirect interaction and solid lines represent direct interaction.

**Figure 3.**
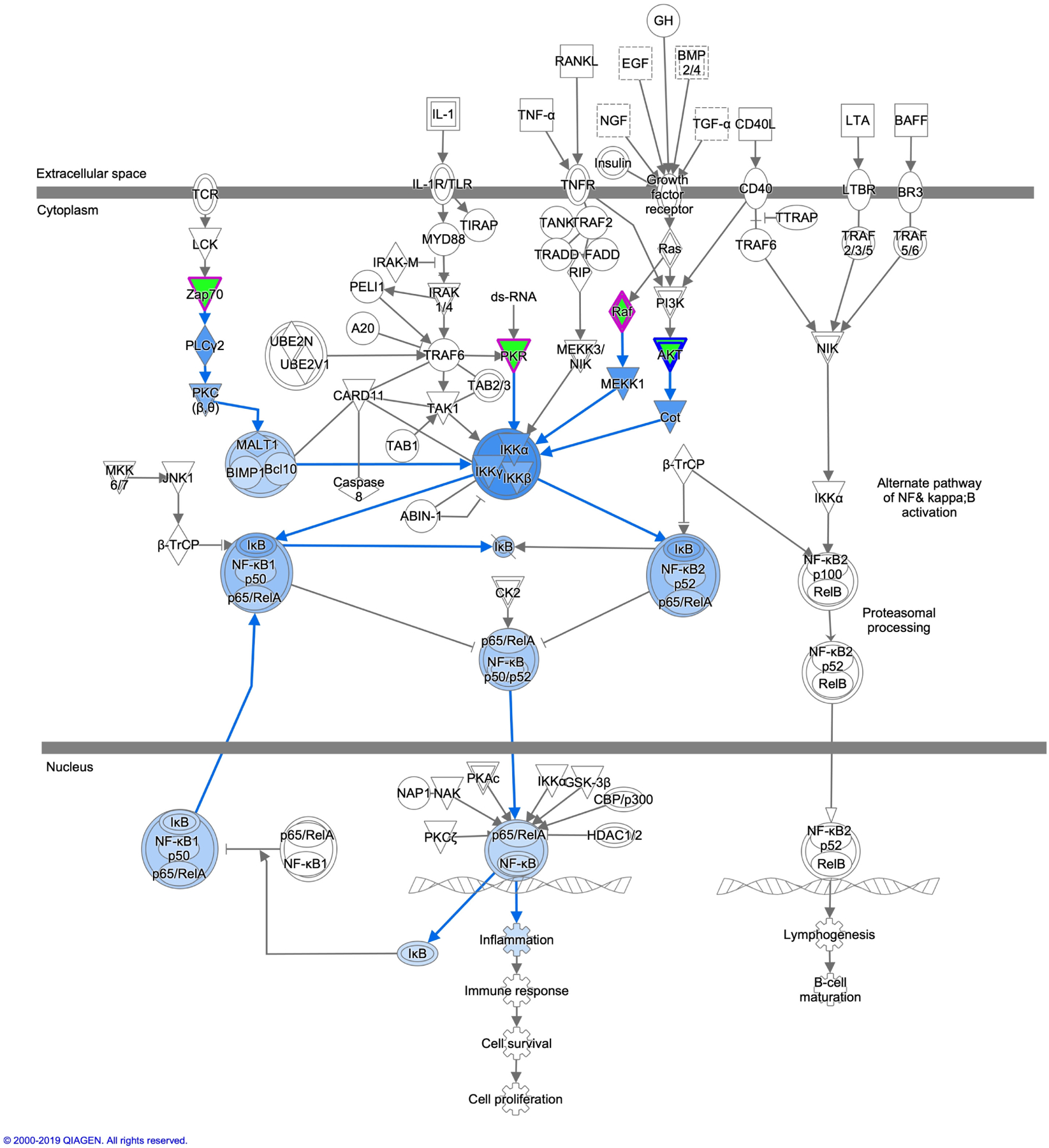
HIV infection affects NF-κB pathway. Four of the 12 kinases (highlighted in green) are involved in NF-κB pathway. HIV-1 modulates NF-κB pathway in order to progress in the transcribe viral mRNA to generate new viral progeny. Modulation of these kinases to decrease NF-κB activity (highlight in blue) may provide a potential therapy.

The antiviral drugs currently on the market are mostly specific for viral proteins. These drugs treat less than 10 human infectious diseases [22] [23]. As previously mentioned the HIV genome is subject to mutations during replication Kinases may provide new drug targets to viral infections including HIV. Through the use of the Ingenuity Pathway Analysis (IPA) software (Qiagen) it is possible to easily analyze interactions of host cell kinases during HIV infection. Upon performing a query of kinases 70 were found to be involved in various stages of HIV infection (Table 1). The top 12 kinases with the most findings were analyzed using the IPA software for where in the HIV infection process are critical and their interrelations ship with each other (Figure 1 and Figure 2). Akt1 appears has multiple kinases directly or indirectly affected its activity. Four of these kinases are involved in NF-κB activation during viral infection and modulation of these kinase activates may be a potential therapy to attenuate the activation of this important pathway for HIV (Figure 3). In the following section we provide a brief background on these 12 kinases and relevant findings to their phenotypes in HIV infection. In addition we provide a table that relates the HIV proteins Nef, Tat and Vif to these kinases (Table 2).

### 2.1. Adenosine Kinase (ADK)

ADK is cytoplasmic kinase found in most organ systems that is essential for homeostatic and metabolic regulation. Dysregulation of ADK is known to contribute to diseases such as diabetes, epilepsy and cancer [24]. Adenosine is known for its protective functions such as regulations of angiogenesis and immune responses [25].

The contribution of ADK during HIV infection has been taking into account by the drug Ribavirin, an ADK agonist. The IPA software identifies a correlation between HIV and ADK. The software numerous finding for clinical trials for patients with HIV infection and Hepatitis C have incorporated Ribarvin, a nucleo-side analogue, in combination with other drugs [26].

### 2.2. AKT1 (Protein Kinase B)

The protein Akt1 (Protein Kinase B) is a serine/threonine kinase that is critical signaling node in eukaryotic cells and human disease. Akt1 interacts with many biological molecules involved in cell growth, survival, proliferation, angiogenesis and metabolism [27]. Protein Kinase B can cross talk to other kinases involved in other canonical pathways such as NF- κB through the phosphorylation of the IKK*α* [28]. Interestingly HIV replication is inhibited in the absence of Akt1 [29].

Zhou *et al.* suggest that cellular metabolism is essential for HIV replication and Akt1 as a critical host factor. siRNA of Akt1 in Hela P4/P5 cells substantially inhibited HIV infection to 35% of control at 48 hours and <10% at 96 hours post infection [30]. Protein Kinase B plays in the reactivation of HIV in CD4+ and monocyte cells and its inhibition occurs in cART treatment [31] [32].

### 2.3. B-Lymphoid Tyrosine Kinase (BLK)

The non-receptor BLK protein normally expressed B-cells and involved in B-cell antigen rector signaling. There is evidence that BLK is an oncogene as ectopic expression is present in malignant T-cells from patients with cutaneous T-cell lymphoma [33].

In a global analysis of molecules involved with early stage HIV-1 replication König *et al.* identified the involvement of BLK. siRNA against BLK showed greater than >80% reduction in HIV infection in HEK293T cells [34].

### 2.4. Cyclin K (CCNK)

CCNK was discovered as a novel protein that is able to rescues survival of yeast during the absence G1 cyclin [35]. CCNK functions as a regulatory unit for CDK9 where this CCNK/CDK9 complex is involved in transcription elongation. CCNK/CDK9 complex is part of the positive transcription elongation factor b (P-TEFb) that phosphorylates RNA Polymerase II to activate processive elongation [36].

During HIV infection CCNK acts in an inhibitory fashion leading to decreased HIV gene expression. Using yeast two-hybrid, immunoprecipitation and colocalization Khan *et al.* identified interaction of CCNK with HIV Nef. Implication of CCCK inhibitory role in HIV infection was shower in overexpression of CCNK and siRNA knockout of CCNK in Jurkat cells where reduced HIV production and enhance viral released respectively [37].

### 2.5. EIF2AK2 (Protein Kinase R)

Protein Kinase R is a serine/threonine kinase that binds to double stranded RNA (dsRNA) through its dsRNA binding domains [38]. Viral produced dsRNA activates PKR for its antiviral response by phosphorylation of eIF2*α* resulting in the inhibition viral protein synthesis [39]. The viral protein TAT and reverse translational inhibition may bind to PKR similarly to that of eIF2*α* [40]. The expression of tumor suppressor p53 inhibited HIV replication due to the subsequent activation of PKR and phosphorylation of TAT [41].

*In vitro* studies of TAR RNA Binding Protein (TRB) indirectly inactivate Protein Kinase R. Depletion of TRBP using siRNA reduces HIV replication and increases phosphorylation of eIF2*α*. This reduction in virus replication is negated when knocking out Protein Kinase R [42]. Clerzius *et al.* highlight the need for a protein complex consisting of TRBP and adenosine deaminase acting on RNA 1 (ADAR-1) that inhibits Protein Kinase R during the HIV infection [43].

### 2.6. G Protein-Coupled Receptor Kinase 2 (GRK2)

GRK2 is a member of the G-protein receptor kinase family of proteins which phosphorylates the agonist-occupied *β*-adrenergic receptors and other proteins such as p. 38 [44] [45]. Current literature indicates GRK2 plays a role in multiple disease related signaling pathways and is a therapeutic target for cancer and inflammation [45].

In a genome-wide siRNA analyses to determine a host-pathogen biochemical network GRK2 was found to decrease productive infection by 80% (20% of control in 96 h b-GAL expression) [30]. In other siRNA knockout studies decreased infection by 54.4% to 64.8% in HEK 293T cells infected with HIV-1 [30] [34].

### 2.7. Hematopoietic Cell Kinase (HCK)

The cytoplasmic tyrosine kinase HCK is expressed in myeloid cells and B-lymphocytes [46] [47]. It enhances secretions of growth factors and pro-inflammatory cytokines and its overexpression has been linked to leukemia and other cancers. HCK also promotes macrophage polarization. In macrophages in tumors, HCK stimulates podosome formation and facilitates extracellular matrix degradation [48]. HIV Nef activates HCK through the SH3 domain produces proinflammatory vesicle release [49] [50].

In order to penetrated mucosal epithelium HIV-1 will infect dendritic cells using the dendritic cell immunoreceptor (DCIR) as a binding factor [51]. In a study using antisense oligonucleotides Lambert *et al.* show that HCK is involved in dendritic cell immunoreceptor (DCIR) mediated HIV-1 entry/binding [52]. Through immunoprecipitation experiments HCK was found to bind with HIV Vif specifically through the SH3 domain. HCK inhibited the production and infectivity of HIV virions in infected cells lacking Vif [53].

### 2.8. Mitogen Activated Protein Kinase Kinase Kinase 11 (MAP3K11)

MAP3K11 is a ubiquitously expressed protein of the serine/threonine kinase family [54]. It is involved in the activation of c-Jun N-terminal Kinase (JNK), p38 MAPK and extracellular signal-regulated kinases 1 and 2 (ERK1/2) [55]. The MAP3K11 protein possesses a SH3 domain that is critical in Hepatitis C infection to prevent MAP3K11 apoptosis [56].

Nguyen *et al.* found MAPK311 is an enhancer to HIV infection. MAP3K11 enhances Tat-dependent transcription 3 fold leading to increased HIV infection signal [57]. SiRNA knockouts of MAPK311 reduced HIV infection in both HelaCD4*β*Gal and Jurkat cells. Data suggests that MLK3 enhances HIV transcription through the AP-1 site located in the LTR region [57].

### 2.9. Macrophage Stimulating 1 Receptor (MST1R)

MST1R is a c-Met receptor tyrosine kinase that serves as cell surface receptor in epithelial cells, osteoclasts, and macrophages for the glycoprotein macrophage stimulating protein (MSP). The binding of MSP to MST1R regulates proliferation, survival, and chemotaxis [58]. MST1R has been found to be overexpressed in a large number of breast cancers leading to a high chance for metastasis. A deficiency of MST1R in mice is linked to toxic shock as macrophages are unable to down-regulate certain pro-inflammatory [58].

In HIV infection MST1R plays a significant role in transcription of proviral DNA. MST1R overexpression in U937 cells decreases the binding of NF-κB to HIV LTR. RNA Polymerase II processivity is paused in the presence of MST1R in HIV infected cells at multiple checkpoint in transcription [59].

### 2.10. P21-Activated Kinase (PAK)-1

PAK-1 is a serine threonine kinase of the group I PAK family first discovered in 1994 in rat brain that binds to the GTPase Cdc42 and Rac1 [60]. In addition to cell function such as cytoskeleton organization, migration and proliferation PAK1 promotes tumor development by prevent apoptosis in cancer cells [61]. HIV Nef activates PAK1 which in turn activates the JNK [62]. The highly related PAK2 is known to be activated by HIV Nef to affect the ERK pathway in T cell lines [63].

Work by Nyugen *et al.* shows PAK-1 is critical kinase for integration of provirus into infected cells. The absence of PAK1 by siRNA reduced integration of provirus into genomic DNA of HIV infected cell while overexpression of active PAK-1 increased viral integration. Depletion of PAK2 did not mirror this phenotype [64].

### 2.11. RAF-1

Raf-1 is serine threonine kinase involved in the ERK activation pathway. After being phosphorylated by Ras, RAF-1 initiated a kinase cascade by phosphorylating MAPK/ERK kinase (MEK) which phosphorylates ERK. Raf-1may also be activated by other kinase such as Protein Kinase C or other tyrosine kinases [65]. The ERK pathway contributes to multiple cell processes including T cell activation [66] RAF-1 is also part of the activation of the pathway [67].

Popik *et al.* investigated the role of Raf-1 in HIV-1 infection. In Jurkat T cells Raf-1 is activated by the binding HIV-1 virions through interactions with the kinase Lck not Ras. Overexpression of active Raf-1 increases HIV-1 replication and HIV-1 promotor activity synergistically with HIV Tat [65]. Gringhuis *et al.* expands on Raf-1 role in HIV-1 replication by identifying the need for Raf1 in dendritic cells during infection. Binding of HIV to inducing DC-SIGN Raf-1 activation plays a critical role in transcription elongation of HIV provirus through pTEF-b-mediated phosphorylation of RNA Polymerase II [68].

### 2.12. Zeta-Chain Associated Protein Kinase 70 (ZAP70)

ZAP70 is a cytoplasmic tyrosine kinase necessary for T-cell antigen receptor (TCR) signaling [69]. ZAP70 possesses two tandem SH2 unit which controls its binding capabilities to immunoreceptor tyrosine-based activation motifs (ITAMs) and has a crystal structure similar to that of CDKs and SRC kinases [69]. Numerous studies identify the importance of ZAP70 in signal transduction from TCR [70]. Mutations in the zap70 gene cause the loss of CD8+ T cells and CD4+ cells that are unresponsive to CD2- and CD3-meditated activation [71] [72] [73].

For the first time Tardif *et al.* describe that it is the contribution of ZAP70 in entry of HIV-1 particles in primary human CD4^+^ T cells. ZAP70 plays an important role in entry of HIV virions bearing host cell membrane protein Inter-cellular Adhesion Molecule (ICAM-1) in CD4+ lymphocytes [74]. Sol-Foulon *et al.* found that T-lymphocytes deficient in ZAP70 activity have impaired HIV replication based on measurement of p24. Interestingly the necessity of ZAP70 was not required in the early stages of HIV infection but delays replications and is prerequired for the formation of viral synapses between cells for transmission [75].

## 3. Conclusion

HIV-1 modulates multiple kinases in the host cell to maintain the lifecycle. The 12 kinases we examined in the review are involved in multiple areas of the lifecycle including HIV binding/entry, provirus integration and replication. These host cell kinases are present in various areas of the cell and directly or indirectly affect the activity of each other. Not surprising many of these kinases affect the activity of Akt1 which plays a major role in metabolism and cell survival. HIV-1 infection requires NF-κB activation for transcription of its proviral genes to complete the lifecycle we have identified four kinases involved in this pathway. Targeting these kinases to attenuate the transcription of HIV genes critical for the lifecycle may be potential therapeutic targets.

## Figures and Tables

**Table 1.** IPA Bioprofiler query displaying 70 kinases involved in HIV infection.

Symbol	Molecule Activity	Effect on Disease or Function	Disease or Function	Causal or Correlated	Findings
**ADK**	increased activity	affects	HIV infection	correlation	17
**MST1R**	increased activity	decreases, increases	Binding of HIV-1	causal	6
**HCK**	decreased activity, increased activity	decreases, increases	Binding of HIV-1, Delay in replication of HIV-1, Infectivity of HIV-1, Production of HIV-1	causal	5
**PAK1**	decreased activity, unknown change in activity	decreases, increases	Infection by HIV-1, Integration of HIV-1	causal	5
**AKT1**	decreased activity, increased activity	decreases, increases	Infection by HIV-1	causal	4
**CCNK**	decreased activity, increased activity	decreases, increases	Replication of HIV-1	causal	4
**RAF1**	decreased activity, increased activity	decreases, increases	Replication of HIV-1, Transmission of HIV-1	causal	4
**EIF2AK2**	decreased activity, increased activity	decreases	Production of HIV-1, Replication of HIV-1	causal	3
**GRK2**	decreased activity	decreases	Infection by HIV-1	causal	3
**MAP3K11**	decreased activity, increased activity	decreases, increases	Infection by HIV-1	causal	3
**ZAP70**	decreased activity	decreases, increases	Delay in replication of HIV-1, Entrance of HIV-1, Transmission of HIV-1	causal	3
**BLK**	decreased activity	decreases	Infection by HIV-1	causal	2
**BMP2K**	decreased activity	decreases	Infection by HIV-1	causal	2
**CHUK**	increased activity	increases	Binding of HIV-1	causal	2
**COASY**	decreased activity	decreases	Infection by HIV-1	causal	2
**DCAF1**	decreased activity, increased activity	decreases, increases	Infection by HIV-1	causal	2
**EXOSC10**	decreased activity	decreases	Infection by HIV-1	causal	2
**FYN**	decreased activity	decreases	Binding of HIV-1, Production of HIV-1	causal	2
**IKBKG**	decreased activity, increased activity	Decreases, increases	Infection by HIV-1	causal	2
**JAK1**	decreased activity	decreases	Infection by HIV-1	causal	2
**LIMK2**	decreased activity	decreases	Infection by HIV-1	causal	2
**MPP2**	decreased activity	decreases	Infection by HIV-1	causal	2
**PANK1**	decreased activity, increased activity	decreases, increases	Infection by HIV-1	causal	2
**PFKL**	decreased activity	decreases	Infection by HIV-1	causal	2
**PFKM**	decreased activity	decreases	Infection by HIV-1	causal	2
**PIKFYVE**	decreased activity, increased activity	affects, decreases	Replication of HIV	causal	2
**PIP5K1C**	decreased activity, increased activity	decreases, increases	Infection by HIV-1	causal	2
**PLK1**	decreased activity	decreases	Infection by HIV-1	causal	2
**PRKAA1**	decreased activity	decreases	Infection by HIV-1	causal	2
**PRKCA**	decreased activity	decreases	Binding of HIV-1, Production of HIV-1	causal	2
**PRKCH**	decreased activity	decreases	Infection by HIV-1	causal	2
**WNK1**	decreased activity	decreases	Infection by HIV-1	causal	2
**BCR**	decreased activity	decreases	Infection by HIV-1	causal	1
**BRDT**	decreased activity	decreases	Infection by HIV-1	causal	1
**CAMK1D**	decreased activity	decreases	Infection by HIV-1	causal	1
**CAMKK2**	decreased activity	decreases	Infection by HIV-1	causal	1
**CCT2**	decreased activity	decreases	Infection by HIV-1	causal	1
**CIB2**	decreased activity	decreases	Infection by HIV-1	causal	1
**CRIM1**	decreased activity	decreases	Infection by HIV-1	causal	1
**DAPK2**	decreased activity	decreases	Infection by HIV-1	causal	1
**DLG1**	increased activity	affects	Replication of HIV-1	causal	1
**DMPK**	increased activity	decreases	Viral release of HIV-1	causal	1
**DYRK1A**	unknown change in activity	affects	Replication of HIV-1	causal	1
**EGFR**	decreased activity	decreases	Infection by HIV-1	causal	1
**ERN2**	decreased activity	decreases	Infection by HIV-1	causal	1
**GCK**	decreased activity	decreases	Infection by HIV-1	causal	1
**ITPKA**	decreased activity	decreases	Infection by HIV-1	causal	1
**LCK**	increased activity	increases	Replication of HIV-1	causal	1
**LIMK1**	decreased activity	decreases	Viral entry by HIV-1	causal	1
**MAP3K14**	decreased activity	decreases	Infection by HIV-1	causal	1
**MAP3K7**	decreased activity	decreases	Infection by HIV-1	causal	1
**MAP3K9**	decreased activity	decreases	Infection by HIV-1	causal	1
**MOS**	decreased activity	decreases	Infection by HIV-1	causal	1
**MTOR**	decreased activity	affects	HIV infection	correlation	1
**MYLK**	increased activity	affects	HIV encephalopathy	correlation	1
**NEK9**	decreased activity	decreases	Infection by HIV-1	causal	1
**NRBP1**	decreased activity	decreases	Infection by HIV-1	causal	1
**PAK3**	decreased activity	decreases	Infection by HIV-1	causal	1
**PANK3**	decreased activity	decreases	Infection by HIV-1	causal	1
**PCK1**	decreased activity	decreases	Infection by HIV-1	causal	1
**PI4KA**	decreased activity	decreases	Infection by HIV-1	causal	1
**PKN2**	decreased activity	decreases	Infection by HIV-1	causal	1
**PRKX**	decreased activity	decreases	Infection by HIV-1	causal	1
**PTK2B**	decreased activity	decreases	Replication of HIV-1	causal	1
**RPS6KA3**	decreased activity	decreases	Infection by HIV-1	causal	1
**SIK1/SIK1B**	decreased activity	decreases	Infection by HIV-1	causal	1
**SRPK3**	decreased activity	decreases	Infection by HIV-1	causal	1
**TAOK1**	decreased activity	decreases	Infection by HIV-1	causal	1
**TNK1**	decreased activity	decreases	Infection by HIV-1	causal	1
**TWF1**	decreased activity	decreases	Infection by HIV-1	causal	1

**Table 2.** Association between HIV proteins and host cell kinases.

HIV Protein	Functions in Virus Lifecycle	Host Cell Kinase Interactions
**Nef**	Downregulates CD4 receptor expression NF-κB Activation T-Cell Activation Increase Tat expression	CCNK HCK PAK1
**Tat**	NF-κB dependent HIV −1 LTR activation Necessary for Replication	RAF 1
**Vif**	Replication in Lymphocytes and Macrophages Inactivation of cytidine deaminases	HCK
